# Novel Insights into the Effects of Different Cooking Methods on Salted Egg Yolks: Physicochemical and Sensory Analysis

**DOI:** 10.3390/foods13131963

**Published:** 2024-06-21

**Authors:** Xuejing Gao, Mengya Zhang, Junhua Li, Luping Gu, Cuihua Chang, Zijian Huang, Yanjun Yang, Yujie Su

**Affiliations:** 1 State Key Laboratory of Food Science and Resources, Jiangnan University, Wuxi 214122, China; 2 School of Food Science and Technology, Jiangnan University, Wuxi 214122, China; 3 College of Bioscience and Biotechnology, Hunan Agricultural University, Changsha 410128, China

**Keywords:** thermal treatment, fatty acid, amino acid, volatile organic compound, sensory profile, electronic nose, electronic tongue

## Abstract

In this study, the flavor characteristics and physicochemical properties of salted egg yolk (SEY) under different cooking methods (steaming/baking/microwaving) were investigated. The microwave-treated SEY exhibited the highest levels of salt content, cooking loss, lightness, and b* value, as well as the highest content of flavor amino acids. A total of 31, 27, and 29 volatile compounds were detected after steaming, baking, and microwave treatments, respectively, covering 10 chemical families. The partial least squares discriminant analysis confirmed that 21 compounds, including octanol, pyrazine, 2-pentyl-furan, and 1-octen-3-ol, were the key volatile compounds affecting the classification of SEY aroma. The electronic nose revealed a sharp distinction in the overall flavor profile of SEY with varying heat treatments. However, no dramatic differences were observed in terms of fatty acid composition. Microwave treatment was identified as presenting a promising approach for enhancing the aroma profile of SEY. These findings contribute novel insights into flavor evaluation and the development of egg products as ingredients for thermal processing.

## 1. Introduction

Salted egg yolk (SEY) is highly regarded for its excellent nutritional value and extensive availability. The production of SEY typically involves a prolonged process of brine soaking and heat treatment, resulting in protein coagulation and the release of lipids [1]. Lipids make up about 62.5% and proteins about 33% of the dry mass of the yolk, both of which are essential precursors for the formation of flavor compounds in eggs [2]. Flavor is a primary food characteristic that combines volatile and non-volatile components and dramatically influences consumer preferences. It can be anticipated that the organoleptic qualities of eggs change dramatically with the introduction of various heating processes [3]. Salt–heat synergistic treatment can significantly improve the original flavor, texture, and microbiological safety of eggs. It is well known that the formation of volatile organic compounds (VOCs) in high-fat and/or high-protein foods can be regulated by the effect of sodium chloride on the oxidation or degradation of lipids and metabolites therein [4].

Over the past decades, microwaves have gained widespread usage in kitchens as an efficient and energy-saving thermal processing technology [5]. Similar to conventional heating, microwaving plays an indispensable role in cooking, drying, baking, and pasteurization [6]. However, microwaving differs from water baths and baking as it involves volumetric/internal heating instead of convection/conduction heating [6]. It has been demonstrated that microwaving can ensure the flavor and taste of ingredients and improve digestibility, which is a healthy heat treatment without causing the loss of polyunsaturated fatty acids with other nutrients [7]. A novel method for the preparation of salted egg yolk flavoring by a combination of enzymatic hydrolysis and microwave radiation has also been reported in a previous work [8]. In particular, a rapid preparation method was established, which can obtain mature SEY in one day and SEY products with different flavor attributes by comparing varying heat treatment processes such as microwaving, steaming, and baking.

The aroma of SEY plays a pivotal role in assessing its sensory quality, which significantly impacts consumer acceptance and preference. A total of 25 compounds are identified using headspace solid-phase microextraction gas chromatogram–mass spectrometry (HS-SPME-GC-MS) in ultrasound-assisted SEY, comprising 10 esters, 5 alcohols (phenols), 3 hydrocarbons, 2 aldehydes, 2 amines, and 3 heterocyclic compounds [9]. Additionally, the VOCs of SEY are investigated using positive and negative pressure ultrasonic rapid curing technology, revealing hexanal, ethanol, and toluene as the primary determinants of flavor characteristics [10]. With the pursuit of palatability and convenience in food and the development of egg processing technologies, it is crucial to enhance the sensory attributes of SEYs, such as aroma, flavor, aftertaste, and overall acceptability [11].

To date, the majority of studies have primarily focused on optimizing the curing method and texture properties of SEY [12,13,14]. However, the influence of different heat treatment methods on its flavor remains inadequately explored. The research of key aroma compounds in SEY using a partial least squares-discriminant analysis (PLS-DA) model and variable importance projection (VIP) method could elucidate the main volatiles produced during the heating process and provide a foundation for SEY flavor evaluation. Therefore, the main objective of this study was to assess the impact of varying cooking methods (steaming, baking, and microwave) on the flavor profile of salted egg yolk to determine the enhancement effect of different heat treatments on egg yolk quality.

## 2. Materials and Methods

### 2.1. Materials

Fresh hen eggs were obtained from a local farm (Wuxi, China). Flour was purchased from Yihai Kerry Co., Ltd. (Shanghai, China). All chemical reagents of analytical grade were obtained from Sinopharm Chemical Reagent Co., Ltd. (Shanghai, China).

### 2.2. Sample Preparation

Rapid preparation process: The eggs were separated and wiped with filter paper to remove the egg white. The whole yolks were cured in a salt mixture (sodium chloride to wheat flour ratio of 1:2, *w*/*w*) at 25 ± 1 °C in the incubator for one day, keeping the yolk film intact and wrapped separately. At the end of the curing process, the powder attached to the SEY was washed with distilled water, followed by different cooking methods. The cooking method referred to the related literature [3,15] and was slightly modified after several preliminary experiments. Baking SEYs: The SEYs were heated in an oven (Sinmag, MB2-622, Wuxi, China). The top and bottom temperatures were set at 160 °C and preheated for 10 min. The whole yolk was wrapped in tinfoil and placed in the oven for 12 min. Microwaving SEYs: The whole yolk was placed in a glass dish and then heated in the microwave oven (Toshiba, ER-SS20CNW, Foshan, China) at 800 W for 60 s. Steaming SEYs: The whole yolk was sealed in a cooking bag and placed in a steamer (Boxun, BXM-30R, Shanghai, China) at 100 °C for 20 min. The center temperature of each yolk obtained with a probe thermometer (15039, Delta Trak, Pleasanton, CA, USA) was kept uniform, and the three samples were sealed until tested.

### 2.3. Physicochemical Properties

#### 2.3.1. Determination of Salt Content and Cooking Loss

The salt content of SEY was determined according to AOAC (Mohr method) [16]. The mass of SEY derived after different processing methods was weighed with an accuracy of 0.01 g to assess the cooking loss. The cooking loss was calculated by the following equation:

$$\mathrm{Cooking}\;\mathrm{loss}\;\left(\%\right)=\frac{\mathrm{weight}\;\mathrm{of}\;\mathrm{SEY}\;\mathrm{before}\;\mathrm{cooking}\;-\mathrm{weight}\;\mathrm{of}\;\mathrm{SEY}\;\mathrm{after}\;\mathrm{cooking}\;}{\mathrm{weight}\;\mathrm{of}\;\mathrm{SEY}\;\mathrm{before}\;\mathrm{cooking}\;}\;\times \;100$$

#### 2.3.2. Determination of Color Difference

Color measurement was determined using an UltraScan Pro1166 spectrometer (HunterLab, Reston, VA, USA). All samples were cut into pieces with a 0.5 cm thickness. The color space system [17] used was CIE-L*a*b* to represent color coordinate values.

#### 2.3.3. Determination of Oil Exudation

According to the method reported by Fletcher et al. [18], the oil exudation was defined as the proportion of free lipid to total lipid content.

### 2.4. Structure Properties

#### 2.4.1. Determination of T_2_ Relaxation Time

T_2_ relaxation time was carried out by using a 23 MHz NMR analyzer (NMI20-015V-I, Niumag Co., Ltd., Suzhou, China) with a sample tube of 25 mm in diameter. Moreover, 1.5 g SEY samples were placed at the bottom of the NMR tube, and the temperature of the LF-NMR instrument was maintained at 32 °C, which was performed using a sequence based on the Carr–Purcell–Meiboom–Gill (CPMG) sequence. The operating parameters for T_2_ were as follows: number of echoes (NECH) = 4500, number sampling (NS) = 16, time echo (TE) = 0.15 ms, time wait (TW) = 3500 ms.

#### 2.4.2. Attenuated Total Reflectance-Fourier Transform Infrared Spectrometry (ATR-FTIR)

The FTIR spectra were obtained with a spectrometer (Nicolet Co., Ltd., Jackson, WI, USA) equipped with an ATR sample cell. Spectra were collected with 4 cm^−1^ resolution and 32 scans. The analysis of samples was carried out in the range of 600~4000 cm^−1^. OMNIC Series software (v8.2, Thermo Scientific, Waltham, MA, USA) was used for data capture and processing. Each group of samples was scanned independently three times.

### 2.5. Analysis of Fatty Acids

Fatty acid methyl ester (FAME) was obtained by esterification of SEY according to the previous method [19]. The GC (Shimadzu GC-2030, Kyoto, Japan) was equipped with a flame-ionization detector and column with dimensions of 100 m × 0.25 mm and a film thickness of 0.20 µm (Shimadzu Rt-2560, Kyoto, Japan). The temperatures of the injector and detector were 250 °C and 260 °C, respectively. Ultra-high-purity helium was used as the carrier gas with a 1.12 mL/min flow rate and a 10:1 split ratio. The temperature of the column was programmed to 120 °C for 5 min and raised to 240 °C at 5 °C/min and held for 15 min at 240 °C. The external standard method was used to quantify the results as a percentage of peak area. Each sample group was analyzed in triplicate.

### 2.6. Analysis of Free Amino Acids

The sample of 1.0 g was added with trichloroacetic acid (5%, *w*/*v*), fixed to 25 mL, and left to stand overnight at 4 °C. After centrifugation at 7000× *g* for 30 min, the supernatant was filtered through a 0.22 μm syringe filter. The filtrate (20 µL) was injected into an automated online derivatization system and analyzed by HPLC (Agilent 1100, Santa Clara, CA, USA) using a Hypersil ODS column (4.6 mm × 250 mm × 5 µm) with a UV detector operating at 338 nm/262 nm and a flow rate of 1 mL/min. The identification and quantification were decided based on the retention time and the peak area of standard compounds, respectively [20].

### 2.7. Multiple Intelligent Sensory Analysis

#### 2.7.1. Determination of Electronic Tongue (E-Tongue)

The taste was measured using the SA402B taste sensing system (Insent Inc., Atsugi City, Japan). Moreover, 10 g of sample was obtained and mixed with 50 mL of distilled water to extract the flavor substances. The mixture was centrifuged at 7000× *g* for 10 min, and the supernatant was collected for E-tongue analysis [21]. The acquisition time for each sample was 120 s, and the analysis was repeated 4 times.

#### 2.7.2. Determination of Electronic Nose (E-Nose)

The analysis of the E-nose was carried out using Heracles II (Alpha MOS, Toulouse, France) as described in the previous report [17]. Moreover, 2.0 g of samples were accurately weighed and put into 20 mL headspace vials at room temperature equilibrium for 1 h. The parameters were set as follows: injection volume was 5000 μL and injector temperature was 250 °C.

#### 2.7.3. Determination of VOCs

VOCs were extracted and analyzed according to published experimental protocols with some modifications [20]. Briefly, 2.0 g of samples were weighed and placed in a headspace vial. Moreover, 10 μL of internal standard (cyclohexanone, 1.0 μg/μL) were injected and then incubated at 60 °C for 10 min with shaking at 500 rpm to achieve a balance of gas in the headspace. The VOCs of SEY were analyzed using an Agilent 8890-5977B gas chromatograph–mass spectrometry (Agilent, Santa Clara, CA, USA). A 50/30 μm DVB/CAR-BOXEN™/PDMS fiber was used for sample desorption in a GC injector at 260 °C for 5 min in splitless mode. The separation procedure was performed using a DB-WAX capillary column (60 × 0.25 mm, 0.25 μm; J&W Scientific, Folsom, CA, USA) with high-purity helium (99.99%, carrier gas), a flow rate of 1.8 mL/min, and a total ion current of 30–500 amu. The compounds were qualitatively determined by the NIST 14 mass spectral database and by comparison of their retention index (RI), which was calculated by running C7-C40 n-alkanes under the above chromatographic conditions.

#### 2.7.4. Sensory Evaluation

A total of 10 panelists (n = 10, aged between 20~30 years old), who had previous experience in sensory evaluation, are from the School of Food Science and Technology, Jiangnan University. All samples were aliquoted into randomly coded containers and presented to the panelists. Each sample was independently presented to the panel three times for evaluation, and it was maintained at 45 ± 5 °C for assessment. The sensory tests were conducted in the same laboratory at the same time, with constant temperature, humidity, and light control. The sensory assessment was based on a 9-point linear scale to determine the SEYs (9 = intense and 1 = mild), such as savory aroma, egg yolk aroma, grease flavor, unpleasant smell, and overall preference. Consent: The sensory panelists were provided with comprehensive information and explicitly consented to the utilization of their evaluation data. The samples used for sensory evaluation consisted of food-grade materials and posed no associated risks. The rights and privacy of all participants were safeguarded throughout the study, with oral consent obtained from expert members.

### 2.8. Statistical Analysis

At least three replicates of the experiment were performed. All data (mean ± SD) were evaluated by Duncan’s multiple range test (SPSS 22.0, SPSS Inc., Chicago, IL, USA) in the analysis of variance ANOVA, and a significant level was considered at *p* < 0.05. The experiment results were plotted using Origin 2023 (Origin Lab Corporation, Northampton, MA, USA) and Simca 14.1 (Sartorius Stedim Data Analytics AB, Umeå, Malmö, Sweden) was calculated for PLS-DA and VIP. Heatmap (hierarchical cluster analysis) was available at https://www.bioladder.cn/web/#/pro/index. Principal component analysis (PCA) and discriminant factor analysis (DFA) of E-nose were performed by AlphaSoft V14 software. The Venn network was available at http://www.ehbio.com/test/venn/. 

## 3. Results and Discussion 

### 3.1. Physicochemical Properties of SEY

#### 3.1.1. Salt Content and Cooking Loss

**Figure 1:**
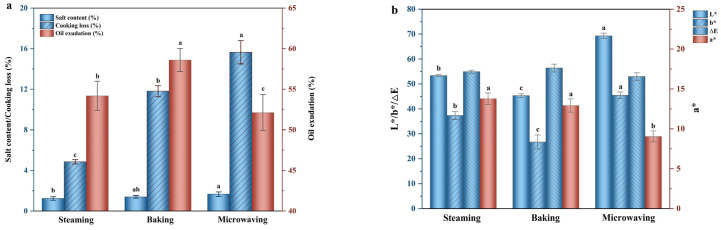

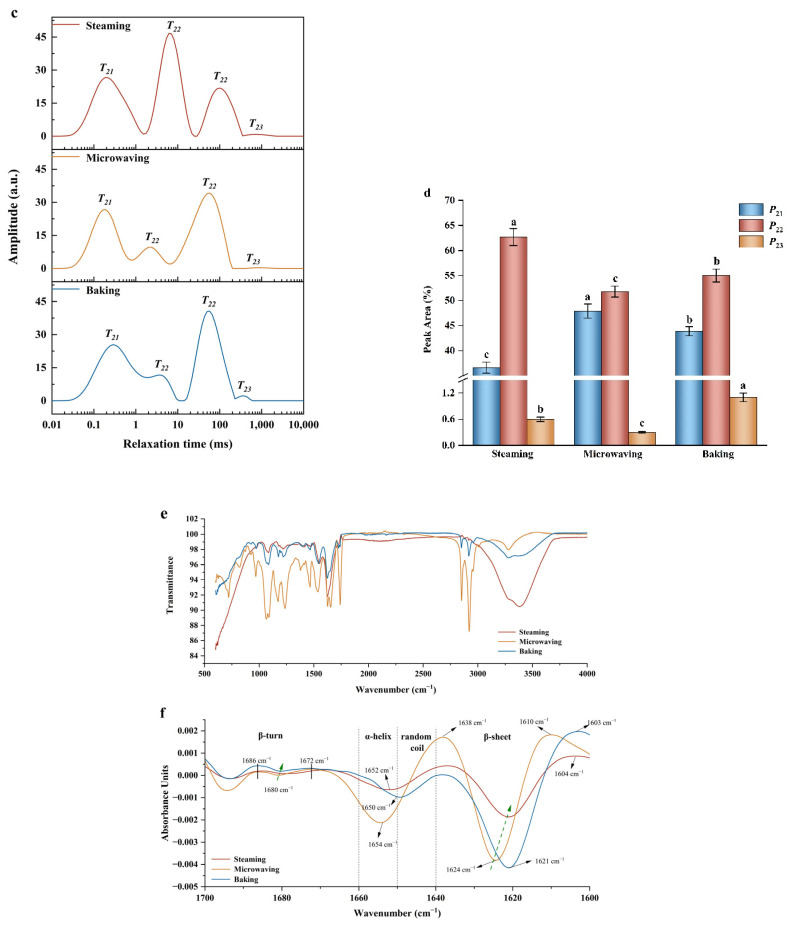
Physicochemical properties analysis of SEY. Salt content, cooking loss, and oil exudation (**a**); instrumental color (L*/a*/b*/∆E) (**b**); LF-NMR relaxation spectrum (**c**); peak area of LF-NMR relaxation spectrum (**d**); ATR-FTIR curves (**e**); secondary structure existed in the amide I (**f**). Different lowercase letters in the Figures indicated significant differences (*p* < 0.05).

The effect of different cooking methods on salt content and cooking loss was intensely related to the sensory quality of the food material. The physicochemical properties of SEY after being processed through different heating treatments were shown in Figure 1a. It can be found that salt content and cooking loss obtained a consistent result as follows: microwaving > baking > steaming. The water-holding capacity of SEYs was assessed through cooking losses, which were attributed to the shrinkage of egg yolk proteins during heating, leading to the release of water molecules. This phenomenon facilitated a greater retention of salt content in SEY with higher water loss for an equivalent mass. In contrast, microwave-treated SEY exhibited rapid heat generation through friction with water molecules, resulting in a swift increase in core temperature and relatively uniform heating. However, it is worth noting that the application of microwave treatment may lead to significant weight reduction, which can adversely impact the economic viability of egg products. Furthermore, previous evidence has demonstrated a close correlation between moisture and food texture, thereby resulting in a significant reduction in the smoothness of SEY after microwave heating [3].

#### 3.1.2. Oil Exudation

Oil exudation is a crucial attribute that reflects the grade and acceptability of SEY, with high-quality SEY being characterized by its richness in oil content [22]. As indicated in Figure 1a, baking resulted in significantly higher oil exudation than steaming and microwave heat-treated methods (*p* < 0.05). Juhaimi et al. [6] observed that various cooking methods significantly impact the oil content. According to previous reports, most of the lipids in egg yolk are present in low-density lipoproteins (LDLs) [23]. During salt-induced processing, free lipids may be released from LDL micelles due to changes in LDL structure caused by dehydration and high salt conditions. The heating process further promoted LDL destruction and denaturation of other proteins responsible for stabilizing lipid content in egg yolk [24]. Therefore, the highest oil exudation in the baking group can be attributed to the elevated heating temperature, which facilitates a more pronounced thermal denaturation process resulting in the separation of yolk lipids from proteins.

#### 3.1.3. Instrumental Color

The color of food has long been considered significantly correlated with its quality, and processing methods play an important role in altering raw material color [25]. The microwave group exhibited the highest L* and b* values in Figure 1b, while the baking and steaming groups showed significantly higher a* values (*p* < 0.05). Furthermore, there was no significant difference observed in ΔE values among the three treatments (*p* > 0.05). Among all samples, baking produced the darkest brown color for SEY due to both high baking temperatures promoting protein disulfide bond polymerization and oxidation reactions, as well as facilitating the Maillard reaction, which leads to browning [17].

#### 3.1.4. T_2_ Relaxation Time

In general, the relaxation time provides insights into the flow state of different water and lipid components, while the peak area serves as an indicator of their content [26]. As shown in Figure 1c, the T_2_ relaxation time spectrum of SEY under different cooking methods consisted of four peaks, where 0~2 ms was attributed to T_21_, 2~350 ms to T_22_, and more than 350 ms to T_23_. They represented water that was tightly bound to proteins, non-mobile water associated with lipid signaling, and the more mobile lipids in egg yolks, respectively [27]. It could be observed that the T_22_ proton signals were stronger in the steaming group than in the microwave and baking groups, which might be attributed to the fact that there were still significant differences in the degree of unfolding of proteins and exposure of mobile water molecules by the different heating methods, although heating causes disruption of the lipoprotein structure and frees the lipid molecules from the proteins. Meanwhile, the T_23_ proton signal was stronger in the baking group, suggesting a potential higher free lipid content in the egg yolks, which was also consistent with the results of oil exudation detected by the conventional method. The percentage of the three peak areas was depicted in Figure 1d, which was a more visual reflection of the water–oil distribution in the samples under the three heating modes. The microwave group exhibited the highest levels of P_21_, potentially attributed to enhanced protein–water and protein–lipid interactions, leading to the conversion of a portion of T_22_ into T_21_.

#### 3.1.5. FTIR Analysis

The ATR-FTIR transmittance spectra, as depicted in Figure 1e, can be attributed to the functional groups present in lipids, proteins, and water within SEYs. There was little difference in the shape of the spectra for the microwave and baking groups, indicating that the chemical composition of their corresponding samples was more similar [28], whereas the differences with the steaming group were more pronounced. Seven bands were observed at 1085 cm^−1^, 1172 cm^−1^, 1236 cm^−1^, 1464 cm^−1^, 1743 cm^−1^, 2850 cm^−1^, and 2925 cm^−1^, all of which were associated with functional groups of the yolk lipid constituents, in agreement with previous results [29]. The amide I region, spanning from wave number 1700 cm^−1^ to 1600 cm^−1^ (Figure 1f), encompasses the most informative spectral range in the FTIR spectra for characterizing protein secondary structure. In particular, the stretching vibrations of the protein carbonyl group (C=O) correspond to positions 1649 cm^−1^, 1652 cm^−1^, and 1654 cm^−1^, where the vibrations of the microwave group were more intense. In contrast, the differences in fluctuations in the b-turn region of the three samples located at 1660 cm^−1^ ~1700 cm^−1^ were not evident. Furthermore, the secondary structure of proteins in SEY was dominated by the β-sheet, followed by the α-helix. Overall, it appeared that the peak shapes and peak positions in the secondary structure spectra of the proteins tended to be consistent, but the intensities varied, suggesting that the internal structural changes of the proteins were affected differently due to the different modes of heating.

### 3.2. Fatty Acid Analysis

A total of 23 fatty acids were detected in all three SEYs, among which 14 representative fatty acids (more than 99% in total) were selected and presented in Table 1. There was no significant difference in the content of oleic acid (C18:1) as the major fatty acid in various heating methods, ranging from 42.66% to 42.94% (*p* > 0.05). Interestingly, linoleic acid (C18:2) showed an opposite trend compared to palmitic acid. The above results were similar to the results reported by Harlina et al. [30]. Egg yolks were known to contain essential unsaturated fatty acids (UFAs), including n-3 polyunsaturated fatty acids (PUFAs) and n-6 PUFAs, which play a vital role in regulating biological activities [31]. The fatty acid distribution of SEY remained consistent regardless of the method used for heat treatment, and there were no significant differences in the composition of SFA and unsaturated fatty acids due to the influence of heat-treated methods (*p* > 0.05). It has been reported that the genetic composition of raw materials and growth conditions are influential factors contributing to significant variations in fatty acid composition [32], while the impact of processing methods on fatty acid composition may be very limited in this study.

### 3.3. Free Amino Acids (FAAs)

It is well known that certain amino acids play an essential role in establishing the flavor profile of foods, some contributing distinct flavor characteristics and others serving as precursors for odors and other flavor compounds [33]. As depicted in Table 2, there existed a statistically significant difference (*p* < 0.05) in the composition of FAAs across different heat treatments applied to SEY. This disparity can likely be attributed to variations in moisture loss caused by various cooking methods during processing. Glutamic acid (Glu) was the most abundant amino acid of the three samples, and it, along with aspartic acid (Asp), had a pleasant fresh taste, often contributing greatly to umami [34]. The ratio of umami amino acids (Glu and Asp) to total amino acids was also consistent with the result of umami by E-tongue analysis. In addition, the synergistic effect between alanine (Ala) and Glu could enhance the umami intensity of foods [34]. Umami constituted the predominant flavor profile of SEY; however, the proportion of sweet amino acids, namely serine (Ser), alanine (Ala), and glycine (Gly), was relatively minimal, accounting for merely 9.14% to 9.51% of the total amino acid composition. On this basis, the results of flavor amino acids and essential amino acids were followed by a similar trend of total amino acids. Interestingly, the changes in histidine (His) and cysteine (Cys-s) exhibited distinct patterns compared to other FAAs. Specifically, no significant variation was observed in the content of His, one of the bitter-tasting amino acids. This finding may elucidate that microwaving and baking were not conducive to the production of bitter amino acids. Meanwhile, microwaves appeared to facilitate the degradation of Cys-s. Above all, heating methods had hardly any effects on most of the FAAs. It was shown from a study demonstrated that cooking did not alter the ratio of essential amino acids to non-essential amino acids in eggs [3], which remained within a range of 76.99% to 79.11%, in agreement with the results concluded in the current work.

### 3.4. Comprehensive Sensory Evaluation

#### 3.4.1. E-Nose Analysis

**Figure 2:**
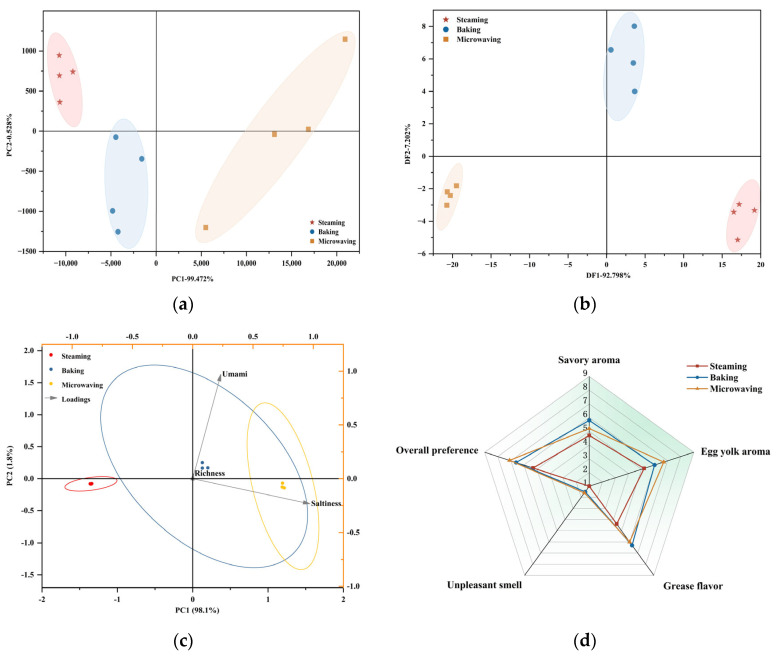
Multi-sensory analysis of SEY. PCA of E-nose (**a**); DFA of E-nose (**b**); PCA of E-tongue (**c**); Aroma profile by quantitative descriptive analysis (**d**).

The E-nose provided universal information on volatile compounds present in samples, making it ideal for analyzing food odors [35]. The PCA results obtained from the E-nose data were shown in Figure 2a, with variance contribution rates of 99.47% for PC1 and 0.53% for PC2, accumulating to 100%. This indicated that the SEYs had rich overall characteristic identification, where the difference on the *x*-axis can explain 99.47% of the comprehensive analysis results. The distribution of the three samples in different quadrants indicated a wide variation in their flavor profiles. There was evidence that volatile molecules interact with the food matrix, leading to changes in aroma composition [36]. Compared to PCA, DFA was an analytical method capable of reducing variation within classifications while extending variation across classifications. In Figure 2b, the contributions of DF1 and DF2 were 92.798% and 7.202%, respectively, with a cumulative contribution of 100%, which fully reflected the reliability of the sample analysis. In addition, it should be noted that the E-nose exhibited limited sensitivity towards SEY’s aroma substances and only captured a fraction of its overall aroma profile [37].

#### 3.4.2. E-Tongue Analysis

The E-tongue is converted from electrical signals to gustatory signals that distinguish the flavors of food, providing a sensitive threshold and eliminating subjectivity associated with sensory evaluation [38]. PCA was conducted on E-tongue for different heating methods as shown in Figure 2c (PC1 and PC2 explained 98.1% and 1.8% of the total variance, respectively). The E-tongue characterization revealed similarities between the baking and microwaving groups while exhibiting sharp differences from the steaming group. Furthermore, saltiness, umami, and richness displayed distinct correlations as they occupied separate quadrants: microwaving and baking yielded significantly higher umami taste than steaming (*p* < 0.05), whereas there was no significant difference in richness among all three methods (*p* > 0.05). Saltiness followed a pattern of microwaving > baking > steaming, which was consistent with the results of the salt content determination. The results showed that the discrimination of SEY under three heat treatments could be effectively facilitated by employing an E-tongue. A previous study also revealed that different sensors of the E-tongue can significantly differentiate between different varieties of egg yolk flavors with distinct separation distances [39].

#### 3.4.3. Sensory Profile

As depicted in Figure 2d, no significant differences were observed in savory aroma and egg yolk aroma among the different samples (*p* > 0.05). Differences in grease flavor (*p* < 0.05) could result from the different heating temperatures assigned by the various cooking methods and could potentially be associated with water loss from the samples. Additionally, both the microwaving and baking groups exhibited significantly higher overall preference than the steaming group (*p* < 0.05), indicating that desirable high temperatures would be more conducive to producing higher organoleptic acceptability of the samples. It is noteworthy that in comparison to the other two groups, all sensory aspects indicated a tendency for the steaming group to exhibit a milder flavor. These findings suggested that differences between sensory data analyzed using instrumentation and sensory assessment results due to differences in flavor thresholds, or detection limits, as well as disparities in sensitivity levels between humans and machines. Nevertheless, the multidimensional assessment was meaningful in exploring flavor differences and consumer sensory acceptance due to the processing modes of SEYs.

### 3.5. Profiles of VOCs

According to Table 3, a total of 31, 27, and 29 VOCs were detected in the three SEY samples treated by steaming, baking, and microwaving, respectively. These VOCs included 15 aldehydes, 9 pyrazines, 3 alcohols, 2 esters, 6 hydrocarbons, and 6 acids, as well as one each of ketone, furan, thiazole, and sulfide. Notably, the baking group exhibited higher levels of acids and alcohols compared to other groups, whereas no alcohols were found in the microwaving group. Acids generally had a high flavor threshold with limited impact on flavor in SEY. It has been suggested that acetic acid is produced as a by-product during the breakdown process of long-chain fatty acids [40]. In addition, hexanoic acid was detected, which can be attributed to the enzymatic synthesis of UFAs via the lipoxygenase pathway. Alcohols mainly originate from the thermal oxidation of lipids and the degradation of carbohydrates with vegetal and aromatic odors [41]. 1-Octen-3-ol was a common compound present in traditionally prepared SEY but not detected during microwave heating.

The microwave-treated SEY had significantly higher fractions of pyrazines compared to the baking group, while pyrazine fractions were absent in the steaming group. Pyrazine was recognized as one of the primary products of the Maillard reaction, formed by the Strecker degradation of leucine, isoleucine, and glycine, possessing robust sensory attributes [42]. A recent study demonstrated that the content of pyrazine accumulated along with the fermentation process, which ultimately affected the burnt, nutty/cocoa aroma of the food and was also a key aroma substance in SEY [43]. Notably, 2.5-dimethyl-pyrazine had a distinctive baking aroma reminiscent of roasted peanuts/potatoes and played a prominent role in both microwaving and baking treatments. Only trace amounts of ketones were found in the microwaving group. As reported previously, ketones were not typically present in fresh eggs and may arise from secondary reactions involving carboxylic acids during processing [44]. Moreover, ketones generally possessed a high odor threshold with minimal impact on flavor perception [45]. Alkanes, which would have a higher odor threshold and a milder effect on flavor, were usually metabolites of triglyceride degradation and oxidation of fatty acids such as linolenic acid [46].

The three groups were predominantly rich in aldehydes (ranging from 29.04% to 57.70%), highlighting their substantial contribution to the overall aroma composition: microwaving > baking > steaming. Aldehydes were generated through Strecker degradation of amino acids or oxidation/derivatization processes affecting fatty acids during food processing. Previous studies have also identified aldehydes as major components potentially responsible for fried egg aromas [47].

### 3.6. Correlation Analysis of Flavor Profiles

To further explore the flavor distinctions among the three heat-treated SEYs, a detailed analysis was conducted on the volatile substance data. In contrast, PLS-DA served as a supervised statistical method for discrimination, different from PCA for characterizing sample classification trends.

**Figure 3:**
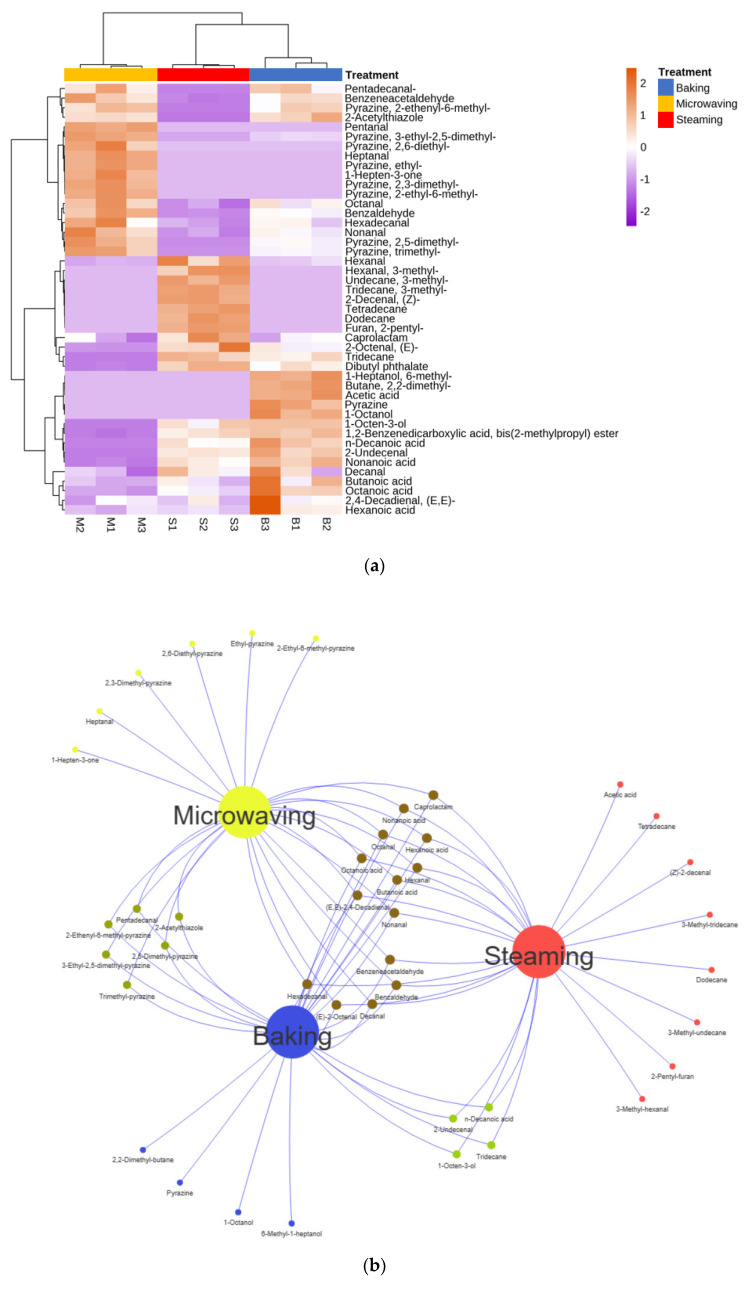

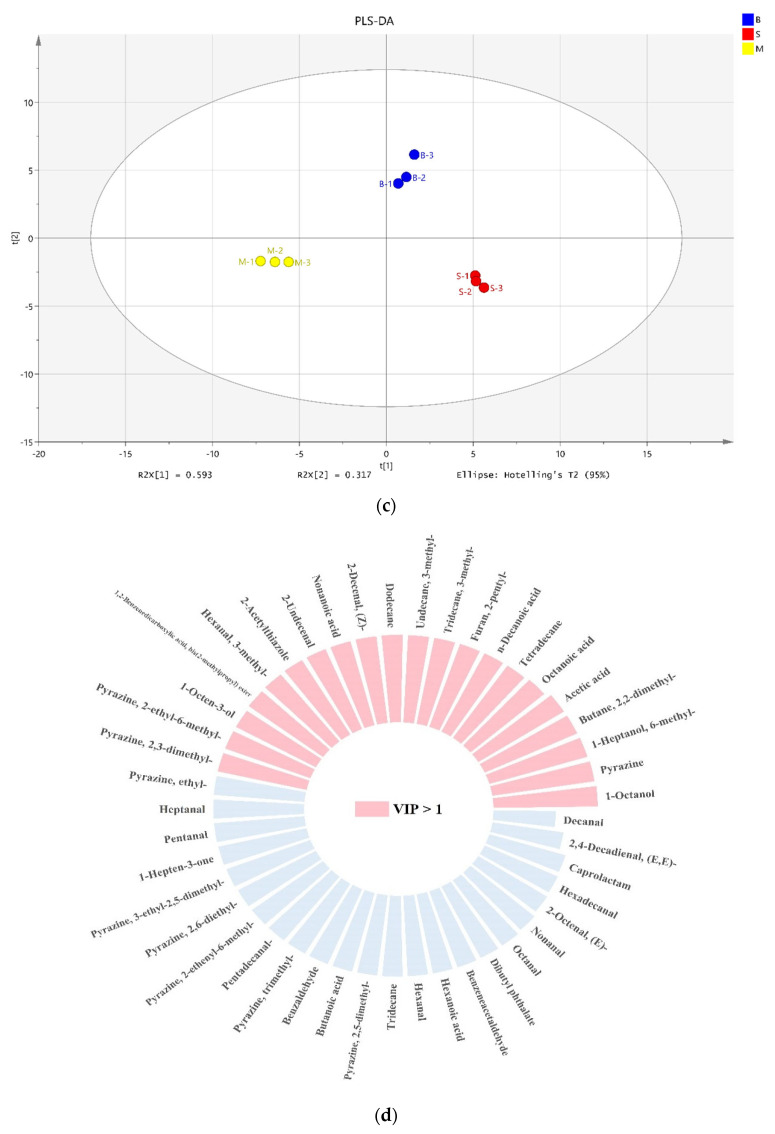
Multiple statistical analyses of VOCs in SEY. Heat map visualization (**a**); Venn diagram (**b**); PLS-DA (**c**); VIP predictive PLS-DA model (**d**).

The correlation analysis of changes in VOCs, using Pearson’s correlation coefficient, was presented in Figure 3a. It can be observed that pentadecanal, benzeneacetaldehyde, pentanal, 3-ethyl-2.5-dimethylpyrazine, 2,6-diethyl-pyrazine, heptanal, ethyl-pyrazine, 1-hepten-3-one, 2,3-dimethyl-pyrazine, 2-ethyl-6-methylpyrazine, octanal, benzaldehyde, hexadecanal, nonanal, 2,5-dimethyl-pyrazine, and trimethyl-pyrazine were positively correlated with the microwaving group. In contrast, hexanal, 3-methyl-hexanal, 3-methyl-tridecane, *(Z)*-2-decenal, tetradecane, dodecane, 2-pentyl-furan, caprolactam, *(E)*-2-octenal, tridecane, and dibutyl phthalate showed positive correlations in the steaming group. Furthermore, the aroma of the baking group and the cooking group exhibited a higher degree of similarity compared to that of the microwave group. The Venn diagram in Figure 3b summarized the SEY volatile compounds produced through rapid preparation under different heat treatments and visually demonstrated the distinct characteristics and variations of aromatic substances. As shown in Figure 3c, a supervised PLS-DA was utilized to examine the differences between the labeled SEY samples [48]. It is evident that all three samples have been clearly distinguished, and PLS-DA has effectively responded to different heating methods of SEY. This finding aligned with results obtained from E-nose analysis. The model exhibited high-grade explained variance (R^2^Y = 0.999) and cross-validated predictive capability (Q^2^ = 0.993), which demonstrated its feasibility. The scores of PC1 (horizontal coordinates) and PC2 (vertical coordinates) were the new variables that summarized the variables. The scores were orthogonal and completely independent of each other. PC1 and PC2 accounted for 59.3% and 31.7% of the total variance, respectively. The scores of PC1 accounted for the highest variation in X-space, followed by PC2. Therefore, the scatterplot of PC1 and PC2 provided insight into how the X observations were interrelated. In order to compare the performance of the PLS-DA models, statistical validation parameters including accuracy, goodness-of-fit (R^2^), and predictive goodness-of-fit (Q^2^) were utilized. The class specificity was excellent, with sensitivities above 90% for all models (R^2^ = 0.91, Q^2^ = 0.971), indicating robust class modeling without any false-positive or false-negative sample classifications [49]. The VIP method was used to explore the characteristic aroma substances in the PLS-DA model with a total sample size of 9 (three varieties in triplicate). The degree of influence and explanatory ability of each variable factor on the classification and discrimination of each group of samples can be assessed by calculating the VIP. As illustrated in Figure 3d, a total of 21 aromatic compounds with VIP values >1 were obtained in the PLS-DA model. A higher VIP value indicated greater differences in aroma compounds between groups that are more important for discriminatory categorization of SEY aroma profiles [37]. Consequently, it can be inferred that volatile compounds such as octanol, pyrazine, 2-pentyl-furan, and 1-octen-3-ol were key components that impacted the categorization of SEY aroma.

## 4. Conclusions

In this study, significant differences in the flavor profile of SEY were observed by different cooking methods, as indicated by E-nose and E-tongue analysis. Both baking and microwaving treatments exhibited higher levels of umami and saltiness. Moreover, a more pronounced correlation was observed among VOCs between the steaming and baking groups, as opposed to the microwave-treated group. Concerning physicochemical characteristics, the microwave-treated SEY exhibited the highest levels of salt content, cooking loss, L* value, and FAAs. Notably, the sensory evaluation indicated a preference for the overall properties of microwave-treated SEY, which suggests the potential of microwaves as an effective way to enhance the flavor of SEY. The discriminant analysis demonstrated the pivotal role of 21 key compounds in determining the classification of SEY aroma, including octanol, pyrazine, 2-pentylfuran, and 1-octen-3-ol. In summary, microwave heating is an appropriate method for achieving the maximum sensory characteristics of SEY. Different heat treatments could provide valuable insights into understanding the variations in SEY flavor components and their impact on physicochemical properties. In the future, it is worthwhile to conduct more in-depth research on functional exploration and nutritional value for varying cooking methods.

## Figures and Tables

**Table 1:** Fatty acid profile (% total fatty acid) according to the different cooking methods of SEY.

Fatty Acids	Steaming	Baking	Microwaving
C14:0	0.26 ± 0.01 ^b^	0.30 ± 0.01 ^a^	0.25 ± 0.01 ^b^
C16:0	25.45 ± 0.08 ^ab^	26.11 ± 0.71 ^a^	24.77 ± 0.33 ^b^
C16:1	3.90 ± 0.49	4.34 ± 0.75	3.37 ± 0.30
C17:0	0.17 ± 0.02 ^a^	0.15 ± 0.01 ^b^	0.19 ± 0.00 ^a^
C17:1	0.16 ± 0.00	0.15 ± 0.01	0.16 ± 0.01
C18:0	8.01 ± 0.28 ^b^	7.92 ± 0.11 ^b^	8.49 ± 0.10 ^a^
C18:1	42.66 ± 1.19	42.94 ± 2.35	42.73 ± 0.27
C18:2	14.76 ± 0.95 ^ab^	13.71 ± 0.66 ^b^	15.82 ± 0.31 ^a^
C18:3n6	0.11 ± 0.01	0.09 ± 0.00	0.09 ± 0.01
C18:3n3	0.34 ± 0.06	0.36 ± 0.04	0.36 ± 0.01
C20:1	0.20 ± 0.01	0.22 ± 0.02	0.21 ± 0.01
C20:2	0.17 ± 0.01	0.18 ± 0.03	0.17 ± 0.00
C20:3	2.57 ± 0.12	2.39 ± 0.18	2.38 ± 0.01
C22:6	1.00 ± 0.18	0.94 ± 0.04	0.79 ± 0.02
Others	0.22 ± 0.03	0.22 ± 0.03	0.20 ± 0.01
Σ SFA ^1^	34.03 ± 0.39	34.59 ± 0.85	33.84 ± 0.44
Σ MUFA ^2^	46.99 ± 1.70	47.73 ± 3.13	46.53 ± 0.59
Σ PUFA ^3^	18.97 ± 1.33	17.68 ± 0.95	19.63 ± 0.36

^1^ Saturated fatty acids. ^2^ Monounsaturated fatty acids. ^3^ Polyunsaturated fatty acids. Different letters in the same row indicated significant differences (*p* < 0.05).

**Table 2:** Composition of free amino acids (mg/100 g) according to the different cooking methods of SEY.

Amino Acids	Steaming	Baking	Microwaving
Asp	40.73 ± 0.67 ^c^	56.08 ± 1.67 ^b^	66.38 ± 1.53 ^a^
Glu	95.33 ± 2.77 ^c^	124.48 ± 5.11 ^b^	150.49 ± 2.66 ^a^
Ser	14.05 ± 1.57 ^c^	17.55 ± 1.78 ^b^	23.34 ± 1.86 ^a^
His	18.18 ± 1.38	19.26 ± 5.78	24.16 ± 0.73
Gly	22.14 ± 0.40 ^c^	27.56 ± 2.72 ^b^	33.13 ± 1.55 ^a^
Thr	53.74 ± 4.61 ^b^	61.38 ± 5.88 ^b^	80.09 ± 8.50 ^a^
Arg	44.95 ± 0.82 ^c^	62.99 ± 1.62 ^b^	76.78 ± 4.53 ^a^
Ala	20.39 ± 0.73 ^c^	26.65 ± 0.46 ^b^	32.78 ± 1.08 ^a^
Tyr	55.77 ± 1.74 ^c^	75.10 ± 1.97 ^b^	84.76 ± 3.27 ^a^
Cys-s	2.50 ± 0.40 ^a^	3.12 ± 0.81 ^a^	1.29 ± 0.28 ^b^
Val	38.49 ± 0.69 ^c^	48.37 ± 2.41 ^b^	57.38 ± 3.06 ^a^
Met	14.30 ± 0.31 ^c^	17.05 ± 0.50 ^b^	21.05 ± 1.56 ^a^
Trp	9.64 ± 0.64 ^b^	10.26 ± 0.28 ^b^	11.52 ± 0.70 ^a^
Phe	28.13 ± 0.52 ^c^	37.65 ± 2.34 ^b^	42.46 ± 1.47 ^a^
Ile	24.66 ± 0.45 ^c^	33.15 ± 1.43 ^b^	40.29 ± 1.55 ^a^
Leu	47.06 ± 1.21 ^c^	65.31 ± 0.96 ^b^	75.92 ± 3.08 ^a^
Lys	49.65 ± 1.04 ^c^	68.22 ± 1.67 ^b^	79.54 ± 5.68 ^a^
Pro	21.64 ± 1.25 ^c^	30.63 ± 1.73 ^b^	37.25 ± 4.84 ^a^
Flavor	262.50 ± 6.82 ^c^	347.51 ± 14.28 ^b^	410.00 ± 11.57 ^a^
Essential	265.66 ± 9.46 ^c^	341.38 ± 15.46 ^b^	408.25 ± 25.60 ^a^
Total	601.36 ± 12.49 ^c^	784.79 ± 13.62 ^b^	938.58 ± 39.96 ^a^

Different letters in the same row indicated significant differences (*p* < 0.05).

**Table 3:** Relative content (ng/g) of VOCs according to the different cooking methods of SEY.

Compound	RI	Steaming	Baking	Microwaving
Acids (6)				
Acetic acid	1449	Nd	9.11 ± 0.21	Nd
Butanoic acid	1625	6.45 ± 0.65 ^ab^	8.09 ± 0.49 ^a^	5.85 ± 0.25 ^b^
Hexanoic acid	1842	3.92 ± 0.36 ^ab^	5.15 ± 0.78 ^a^	3.31 ± 0.23 ^b^
Octanoic acid	2054	2.74 ± 0.09 ^b^	5.06 ± 0.25 ^a^	2.59 ± 0.14 ^b^
Nonanoic acid	2155	61.62 ± 3.72 ^b^	85.15 ± 4.34 ^a^	12.62 ± 1.48 ^c^
Decanoic acid	2271	4.15 ± 0.18	6.62 ± 0.26	Nd
Alcohols (3)				
6-Methyl-1-heptanol	1226	Nd	3.43 ± 0.09	Nd
1-Octen-3-ol	1450	10.85 ± 0.62	14.19 ± 1.36	Nd
1-Octanol	1557	Nd	2.22 ± 0.17	Nd
Aldehydes (15)				
Pentanal	969	Nd	Nd	47.92 ± 2.41
Hexanal	1081	60.55 ± 2.85 ^a^	37.75 ± 1.34 ^b^	35.86 ± 1.19 ^b^
3-Methyl- hexanal	1183	1.05 ± 0.28	Nd	Nd
Heptanal	1188	Nd	Nd	10.18 ± 0.28
Octanal	1287	6.74 ± 0.19 ^c^	10.49 ± 0.45 ^b^	15.65 ± 0.63 ^a^
Nonanal	1392	27.36 ± 1.84 ^c^	45.11 ± 2.80 ^b^	66.48 ± 2.40 ^a^
*(E)*-2-Octenal	1413	9.24 ± 0.41 ^a^	8.86 ± 0.23 ^a^	4.25 ± 0.12 ^b^
Decanal	1495	3.72 ± 0.07	3.82 ± 0.16	3.57 ± 0.06
Benzaldehyde	1518	12.24 ± 0.24 ^c^	35.86 ± 2.61 ^b^	61.87 ± 3.71 ^a^
Benzeneacetaldehyde	1631	4.87 ± 0.08 ^b^	14.13 ± 0.63 ^a^	15.86 ± 0.57 ^a^
*(Z)*-2-Decenal	1640	2.54 ± 0.14	Nd	Nd
2-Undecenal	1712	2.45 ± 0.12	2.59 ± 0.11	Nd
*(E,E)*-2,4-Decadienal	2143	2.55 ± 0.10	2.76 ± 0.26	2.54 ± 0.19
Hexadecanal	2149	6.57 ± 0.17 ^b^	10.16 ± 0.30 ^a^	10.93 ± 0.62 ^a^
Pentadecanal	2242	Nd	2.41 ± 0.12	2.43 ± 0.14
Alkanes (6)				
3-Methyl-undecane	1154	10.29 ± 0.26	Nd	Nd
2,2-Dimethyl-butane	1205	Nd	7.50 ± 0.14	Nd
Dodecane	1201	10.41 ± 0.31	Nd	Nd
Tridecane	1302	8.83 ± 0.21	7.38 ± 0.19	Nd
3-Methyl-tridecane	1369	4.91 ± 0.05	Nd	Nd
Tetradecane	1403	6.48 ± 0.14	Nd	Nd
Esters (2)				
1,2-Benzenedicarboxylic acid, bis(2-methyl propyl) ester	2590	50.34 ± 1.17 ^b^	60.59 ± 2.75 ^a^	7.76 ± 0.33 ^c^
Dibutyl phthalate	2684	75.32 ± 2.14 ^a^	50.42 ± 1.81 ^b^	4.73 ± 0.15 ^c^
Furans (1)				
2-pentyl-furan	1236	25.55 ± 0.57	Nd	Nd
Ketones (1)				
1-Hepten-3-one	1307	Nd	Nd	5.09 ± 0.22
Pyrazines (9)				
Pyrazine	1211	Nd	2.49 ± 0.10	Nd
2,5-Dimethyl-pyrazine	1315	Nd	45.27 ± 2.02	99.73 ± 3.99
Ethyl-pyrazine	1334	Nd	Nd	2.48 ± 0.05
2,3-Dimethyl-pyrazine	1340	Nd	Nd	2.53 ± 0.08
2-Ethyl-6-methyl-pyrazine	1379	Nd	Nd	2.54 ± 0.07
Trimethyl-pyrazine	1398	Nd	10.28 ± 0.11	10.38 ± 1.05
3-Ethyl-2,5-dimethyl- pyrazine	1437	Nd	3.70 ± 0.08	15.20 ± 0.23
2,6-Diethyl-pyrazine	1433	Nd	Nd	3.60 ± 0.18
2-Ethenyl-6-methyl- pyrazine	1504	Nd	2.53 ± 0.13	3.64 ± 0.11
Thiazoles (1)				
Caprolactam	2196	4.89 ± 0.17 ^a^	4.52 ± 0.23 ^a^	2.42 ± 0.18 ^b^
Sulfides (1)				
2-Acetylthiazole	1642	Nd	4.83 ± 0.16	3.70 ± 0.12

Nd = not detected. Different letters in the same row indicated significant differences (*p* < 0.05).

## Data Availability

The original contributions presented in the study are included in the article, further inquiries can be directed to the corresponding author.
